# Editorial: Hemostasis in ECMO and VAD, volume II

**DOI:** 10.3389/fmed.2023.1251079

**Published:** 2023-07-21

**Authors:** Jun Teruya, Jean M. Connors, Barbara Zieger

**Affiliations:** ^1^Transfusion Medicine and Coagulation, Baylor College of Medicine, Texas Children's Hospital, Houston, TX, United States; ^2^Hematology, Harvard Medical School, Brigham and Women's Hospital, Boston, MA, United States; ^3^Division of Pediatric Hematology and Oncology, Department of Pediatrics and Adolescent Medicine, Faculty of Medicine, Medical Center-University of Freiburg, University of Freiburg, Freiburg im Breisgau, Germany

**Keywords:** ECMO, VAD, review, ECPR, bivalirudin

In 2019, first series of *Hemostasis in ECMO and VAD* was published in Frontiers in Medicine—Hematology that included six articles. As 4 years have passed since then, we have updated the series with eight articles addressing various aspects of extracorporeal membrane oxygenation (ECMO) and ventricular assist device (VAD) use. Conditions for use of ECMO such as sepsis and bridge to heart or lung transplant have been more commonly applied and has become a standard of care. ECMO cardiopulmonary resuscitation (ECPR) was initially used for selected patients with cardiac diseases; however, it is more widely used for patients who have cardiac arrest in the hospital or outside the hospital to save their lives regardless of the underlying condition. Recently, increasing number of institutions use bivalirudin as an anticoagulant for ECMO. ECMO is also used for patients with coronavirus diseases 2019 (COVID-19) for recovery from respiratory failure or bridge to lung transplant. In the setting of COVID-19, the duration of ECMO support for these patients was significantly longer such as months to more than a year. Use of VAD is also increasing, especially for the pediatric population, as the wait time for heart transplant is longer than adults. Moreover, some patients on VAD cannot be listed for heart transplant for certain underlying conditions, social reasons, HLA sensitization, or morbid obesity. For these indications, safe long-term anticoagulation and antiplatelet strategies are imperative. This updated series of articles provides the latest information in this area.

Valdes et al. reported that bivalirudin might be a safe and cost-effective alternative to heparin in pediatric ECMO patients after review of seven articles. They concluded that use of bivalirudin as an anticoagulant for ECMO may reduce bleeding, transfusion requirements, and thrombosis with no difference in mortality. They suggested that multicenter studies are needed to compare bivalirudin vs. bivalirudin for ECMO anticoagulation.

Neunert et al. also reported use of the direct thrombin inhibitors, focusing on bivalirudin, for pediatric ECMO. They compared the features of bivalirudin and heparin. They concluded that despite the limited data, there appears to be increasing evidence that bivalirudin may at least be an equally efficacious. Randomized controlled trials are extremely difficult in this diverse population of patients, continued data collection on the safety and efficacy of the use of bivalirudin in ECMO will be required.

Wiegele et al. reported use of enoxaparin for patients with COVID-19 on veno-venous (VV) ECMO compared with unfractionated heparin. They concluded that enoxaparin was superior to unfractionated heparin in terms of thromboembolic primary and the hemorrhagic secondary endpoint by the retrospective findings.

Olson et al. reported their experience in ECPR from a single center. There is growing evidence that ECPR improves survival and neurological outcomes in both in-hospital cardiac arrest and out-of-hospital cardiac arrest. Authors concluded that further randomized clinical trials are needed to identify the most appropriate ECPR candidates and optimal practice in order to conserve limited resources.

Kalbhenn and Zieger stated the importance of “Treat Before They Bleed” approach and minimizing anticoagulation during VV ECMO. It is a strong and useful message for all clinicians that manage ECMO. They also emphasized the importance of the good availability of a coagulation laboratory.

Regling et al. reported use of viscoelastic testing (VET) such as TEG™ and ROTEM™ for pediatric ECMO and VAD. It is not clear if VET alone is enough to monitor hemostasis and anticoagulation effect without conventional plasma based coagulation testing. Since studies of high quality data are limited, the authors concluded that further studies are needed.

Opfermann et al. reported a prospective observational study on Multiplate™, ROTEM™, and thrombin generation assay (TGA) for left VAD. They showed that thrombin receptor activating peptide (TRAP)-induced platelet response on Multiplate™ and ROTEM™ in addition to the standard coagulation parameters might identify patients with platelet dysfunction and alterations of the primary hemostasis. However, TGA parameters were not different in total blood loss among groups of bleeding vs. non-bleeding.

Schlagenhauf et al. reported that Heartmate 3 showed less thrombin generation than Heartmate II with comparable anticoagulation given. They concluded that thrombin generation assay is a useful tool to monitor the coagulation state of patients on VAD and guide anticoagulation.

As the editors of the second series of *Hemostasis in ECMO and VAD*, we are hoping that this series will be useful guides for daily clinical management on ECMO or VAD and future research.

## Author contributions

JT wrote the text. JC and BZ reviewed the text and edited. All authors contributed to the article and approved the submitted version.

